# Aortic Valve Repair with External Annuloplasty in Bicuspid versus Tricuspid Aortic Valve Patients

**DOI:** 10.3390/jcdd11010017

**Published:** 2024-01-06

**Authors:** Davor Baric, Nikola Sliskovic, Gloria Sestan, Savica Gjorgjievska, Daniel Unic, Marko Kusurin, Josip Varvodic, Zrinka Safaric Oremus, Ivana Jurin, Nikola Bulj, Dubravka Susnjar, Igor Rudez

**Affiliations:** 1 Department of Cardiac and Transplant Surgery, Dubrava University Hospital, 10 000 Zagreb, Croatia; nsliskovic@kbd.hr (N.S.); gsestan@kbd.hr (G.S.); sgjorgjiev@kbd.hr (S.G.); dunic@kbd.hr (D.U.); mkusurin@kbd.hr (M.K.); jvarvodi@kbd.hr (J.V.); dubravka.susnjar@gmail.com (D.S.); rudi@kbd.hr (I.R.); 2 Department of Anesthesiology, Reanimatology and Intensive Care Medicine, Dubrava University Hospital, 10 000 Zagreb, Croatia; zsafaric@kbd.hr; 3 Department of Cardiovascular Diseases, Dubrava University Hospital, 10 000 Zagreb, Croatia; ivanajurin1912@gmail.com; 4 Department of Cardiology, University Hospital Centre “Sestre Milosrdnice”, 10 000 Zagreb, Croatia; nikolabulj@gmail.com

**Keywords:** bicuspid aortic valve (BAV), tricuspid aortic valve (TAV), valve-sparing root remodeling (VSRR), aortic valve repair, external annuloplasty, AVIATOR registry

## Abstract

Surgical repair for regurgitant bicuspid aortic valve (BAV) is promising but underutilized due to perceived complexities and lack of long-term data. This study evaluated the efficacy of valve-sparing root remodeling (VSRR) or isolated valve repair combined with calibrated external ring annuloplasty in BAV versus tricuspid aortic valve (TAV) patients. All patients operated on for aortic regurgitation and/or aneurysm at our institution between 2014 and 2022 were included and entered into the Aortic Valve Insufficiency and ascending aorta Aneurysm InternATiOnal Registry (AVIATOR). Patients with successful repair at index surgery (100% in the BAV group, 93% in the TAV group, *p* = 0.044) were included in a systemic follow-up with echocardiography at regular intervals. Among 132 patients, 58 were in the BAV (44%) and 74 in the TAV group (56%). There were no inter-group differences in preoperative patient characteristics, except BAV patients being significantly younger (47 ± 18 y vs. 60 ± 14 y, *p* < 0.001) and having narrower aortic roots at the level of sinuses (41 ± 6 mm vs. 46 ± 13 mm, *p* < 0.001) and sinotubular junctions (39 ± 10 mm vs. 42 ± 11, *p* = 0.032). No perioperative deaths were recorded. At four years, there was no significant difference in terms of overall survival (96.3% BAV vs. 97.2% TAV, *p* = 0.373), freedom from valve reintervention (85.2% BAV vs. 93.4% TAV, *p* = 0.905), and freedom from severe aortic regurgitation (94.1% BAV vs. 82.9% TAV, *p* = 0.222). Surgical repair of BAV combined with extra-aortic annuloplasty can be performed with low perioperative morbidity and mortality and excellent mid-term results which are comparable to TAV repair.

## 1. Introduction

With an incidence of almost 2% in the general population, bicuspid aortic valve (BAV) is the most common congenital cardiac malformation [1]. Due to its common association with abnormalities of the ascending aorta, it should be considered a disease of the entire aortic root. Even though aortic stenosis is a more common presentation of the BAV pathology [1], aortic regurgitation (AR), reported in approximately 20% of these patients, is a non-negligible clinical issue, due to its earlier presentation, most commonly in the third decade of life [2]. Although initially designed for tricuspid aortic valve (TAV), repair techniques have evolved continuously so that in the current guidelines, repair of a leaking BAV falls under the same criteria for repair as TAV—in selected patients, depending on valve anatomy and tissue characteristics, at a comprehensive valve center [3]. Opposed to TAV, anatomical reasons for AR in BAV patients can include a combination of aneurysmal dilatation with distortion of root geometry and primary leaflet pathology, and as such may require a more complex leaflet repair. In spite of reports demonstrating excellent durability and low incidence of recurrent regurgitation [4], BAV repair has still not been widely accepted. Comparable to mitral valve repair, performing annuloplasty increases the rate of successful repairs, especially in the case of a bicuspid valve [5,6,7]. Calibrated external ring annuloplasty is recommended depending on the root anatomy/type of surgery at the sub- and supravalvular levels to restore and enforce the physiologic root anatomy. In this study, we analyzed the results of aortic valve repair for BAV vs. TAV patients using external ring annuloplasty. The primary endpoints of this study were the incidences of AV reintervention and postoperative AR > 2+ and cumulative survival during follow-up. Secondary endpoints were perioperative morbidity and mortality and analysis of echocardiographic parameters on follow-up.

## 2. Materials and Methods

### 2.1. Patient Selection and Study Design

An analysis of prospectively collected data of consecutive patients treated at our institution from 2014 to 2022, who had surgery involving the aortic root and/or aortic regurgitation and were enrolled in the AVIATOR database, was performed [8]. Included were patients with repair of a regurgitant bicuspid or tricuspid aortic valve with or without concomitant aortic root intervention. Excluded were patients with aortic valve/root replacement and patients with unicuspid or quadricuspid aortic valves.

### 2.2. AVIATOR Database

The AVIATOR database/initiative, a part of the Heart Valve Society (HVS) Aortic Valve database, is a multicentric longitudinal observational cohort study enrolling patients with ascending aorta aneurysm and/or AR [8]. It focuses on patients with disorders of the ascending aorta (including the root and supracoronary aorta) and/or isolated aortic regurgitation (including mixed congenital aortic valve disease) to gather data and experiences in order to evaluate the guidelines for surgical indication as well as the use of repair versus replacement with special focus on long-term patients’ outcomes.

### 2.3. Ethics Statement

The local Institutional Review Board’s opinion was requested. They decided that the follow-up was not a medical experiment, and therefore, their approval was not required.

### 2.4. Operative Technique 

Valve-sparing root replacement (VSRR) by means of aortic root remodeling with external ring annuloplasty was previously described and popularized by Lansac [9]. All patients were operated on through full median sternotomy. After establishing a cardiopulmonary bypass, the heart was arrested, and the aorta was cut open with a vertical incision. The height of each cusp was measured with a dedicated caliper [10] to confirm the reparability of the valve. After confirming suitability for the valve-sparing procedure, the sinuses of Valsalva were resected, coronary buttons detached, and external dissection down to the base of the aortic annulus was performed. The size of the LVOT was measured with a Hegar dilator in order to determine the sizes of the subvalvular CORONEO Extra-Aortic ring (Coroneo, Inc., Montreal, QC, Canada) and Valsalva graft (Gelweave Valsalva; Vascutek Ltd., Renfrewshire, UK), respectively. Five pledgeted Ethibond 2-0 sutures (Ethicon Inc., Somerville, MA, USA) were placed on a circumferential plane around the LVOT, 2 mm below the lowest point of insertion of each cusp and at the base of the interleaflet triangles below each commissure. An additional non-pledgeted suture was positioned outside of the LVOT between the right and non-coronary commissure (to avoid conduction disturbances). An appropriate Valsalva graft was then prepared to re-create 2 sinuses in BAV and 3 sinuses in TAV. In BAV patients, sinuses with raphe were merged into a single sinus. The Valsalva graft was anastomosed with a 5-0 Prolene suture (Ethicon Inc., Somerville, MA, USA). Either a flexible prosthetic CORONEO Extra-Aortic ring or a dacron ring was then placed externally and fixed by the previously placed annular Ethibond sutures. At this point, residual cusp prolapse was assessed by repeated measuring of the effective height with a caliper. When the cusp height was inadequate (less than 9 mm), additional cusp repair was performed, predominantly using the central cusp plication technique. Coronary buttons were implanted next, with a 5-0 Prolene suture in standard fashion, and distal anastomosis was performed at the appropriate level of the ascending aorta or arch, depending on the ascending aorta size. 

In cases where the annulus and ascending aorta are within normal dimensions, isolated aortic insufficiency repair with external ring annuloplasty is performed. The aorta is transversely opened 1 cm above STJ, and the quality of valve leaflets, presence of calcifications, fenestrations, and/or retraction of leaflets are assessed to determine the feasibility of repair. Cusps are deemed suitable for repair if the geometric height is ≥17 mm in a tricuspid aortic valve and ≥20 mm in a bicuspid non-fused cusp. To determine the right size of the extra-aortic ring, a Hegar dilatator is used to determine the size of the native aortic annulus, and an appropriate ring size is chosen. Next, an external dissection down to the base of the aortic annulus is performed with careful dissection of the proximal portion of LM and RCA to facilitate the placement of the Dacron extra-aortic open ring. Five pledgeted Ethibond 2-0 sutures (Ethicon Inc., Somerville, MA, USA) are placed on a circumferential plane around the LVOT, with an additional non-pledgeted suture positioned outside of the LVOT between the right and non-coronary commissure in the same fashion as for valve-sparing root replacement. The Dacron extra-aortic ring is cut open and pulled under LM and RCA, then fixated at the ends with a Prolene 4-0 suture following fixation to the annulus by previously placed Ethibond sutures. Cusp prolapse is then assessed by measuring the effective height with a caliper, and if cusp height is inadequate (less than 9 mm for TAV or 10 mm for BAV), additional cusp repair is performed by placing stitches on the free edge of the culprit leaflet until an effective height of 9 mm/10 mm is obtained. For the second ring at the level of the ST junction, a CORONEO Extra-Aortic ring is used, with the same diameter as the annular Dacron ring. At this level, the ring is secured in place with a single 4-0 Prolene suture at each commissure and an additional suture above the orifice of RCA and LM. Transection of the aorta is sutured to the tubular ascending aorta with a standard running suture.

A transesophageal echo was performed to evaluate valve function and repair success.

### 2.5. Follow-Up

Patients with successful repair at index surgery were included in the follow-up. Since the patients were enrolled in the AVIATOR database, they required systematic follow-up with echocardiography at regular intervals. Follow-up was achieved by gathering echocardiographic data using scheduled echocardiography at our institution or from patients by phone or email if all parameters were available in outside-hospital ECHO reports. Aortic annulus measurements were obtained from transthoracic or transesophageal echocardiography using either two-dimensional or three-dimensional methods. The degree of AR was assessed in accordance with the American Society of Echocardiography (ASE) [11].

### 2.6. Statistical Analysis

Continuous variables are expressed as median and interquartile range (IQR). Categorical data are presented as counts and percentages throughout the manuscript. The distribution of continuous variables was controlled by means of the Shapiro–Wilk test. Continuous and discrete variables were compared using a two-sample *t*-test or Mann–Whitney test, where appropriate. Categorical and ordinal variables were compared using Pearson’s Chi-squared test or Fisher’s exact test, where appropriate. The probability of freedom from the event was calculated according to the Kaplan–Meier method. Freedom-from-event curves were compared by log-rank tests. A *p*-value < 0.05 was considered to indicate statistical significance. Statistical analysis was performed using the IBM^®^ SPSS^®^ Statistics software program (version 23.0.0.0 for MS Windows, IBM Corporation, Armonk, NY, USA).

## 3. Results

### 3.1. Patient Cohort

A total of 132 patients undergoing surgery for aortic regurgitation were analyzed. Fifty-eight (44%) had bicuspid aortic valve (BAV group) and seventy-four (56%) had tricuspid aortic valve (TAV group). Demographic data are presented in Table 1. Patients with BAV had a lower incidence of arterial hypertension (66% vs. 87%, *p* = 0.006) and were significantly younger (47 ± 8 years vs. 60 ± 14 years, *p* < 0.001), resulting in significantly lower EuroSCORE I and II scores. 

On preoperative echo, BAV patients had a slightly larger aortic annulus (29 ± 5 mm vs. 28 ± 5 mm, *p* = 0.014), smaller sinus of Valsalva (41 ± 6 mm vs. 46 ± 13 mm, *p* = 0.001), and smaller sinotubular junction diameter (39 ± 10 mm vs. 42 ± 11 mm, *p* = 0.032). There was no significant difference in aortic regurgitation grades, left ventricular diameters, and ejection fraction between groups.

### 3.2. Operative Details

Operative data are summarized in Table 2. An external annuloplasty ring was used in all BAV patients and in 85% of TAV patients (*p* = 0.009). There was a higher percentage of isolated valve repair in the BAV group (22% vs. 9%, *p* = 0.039) and consequently, a higher percentage of sinotubular junction rings used in the BAV group (15% vs. 4%, *p* = 0.004). Significantly more (*p* = 0.003) valve cusp repairs were needed in the BAV group (97%) than in the TAV group (80%). The procedure was more time demanding in the TAV group than in the BAV group, with longer cardiopulmonary bypass (145 ± 46 min vs. 127 ± 40 min, *p* < 0.001) and aortic cross-clamp times (112 ± 32 min vs. 104 ± 24 min, *p* = 0.004). Valve repair success at index surgery was 100% in the BAV group and 93% in the TAV group (*p* = 0.044). 

In one case, repair failed due to calcification of the non-coronary cusp (NCC), despite the fact that decalcification was performed. The second case failed because of multiple fenestrations of all three cusps. In two patients, primary cusp repair was not performed due to the normal appearance of the valve and central jet of AV regurgitation. After the proven incompetence of the aortic valve, during the second cross-clamp, plication of all three cusps was performed but without effect on the final result. Furthermore, in the last case, plication of all three cusps was primarily performed, but massive aortic regurgitation remained. Due to unsuccessful repair, aortic valve replacement was performed in these patients.

### 3.3. Clinical Outcomes

Both groups were analyzed for postoperative complications, which included reopening for bleeding, permanent pacemaker implantation, and cerebrovascular accident (CVA). The data are presented in Table 3. There was no difference between groups. There were no patient deaths in the perioperative period (30 days after surgery or during the same hospitalization) in either group.

### 3.4. Follow-Up

A total of 127 patients out of 132 operated patients (96%) with successful valve repair at index surgery were included in the follow-up. The median follow-up period was 483 days (IQR 946 days). The follow-up analysis contains data on mortality, freedom from valve reintervention, and echo control echocardiography from all (100%) patients included in the study (Table 4).

### 3.5. Survival Analysis

**Figure 1:**
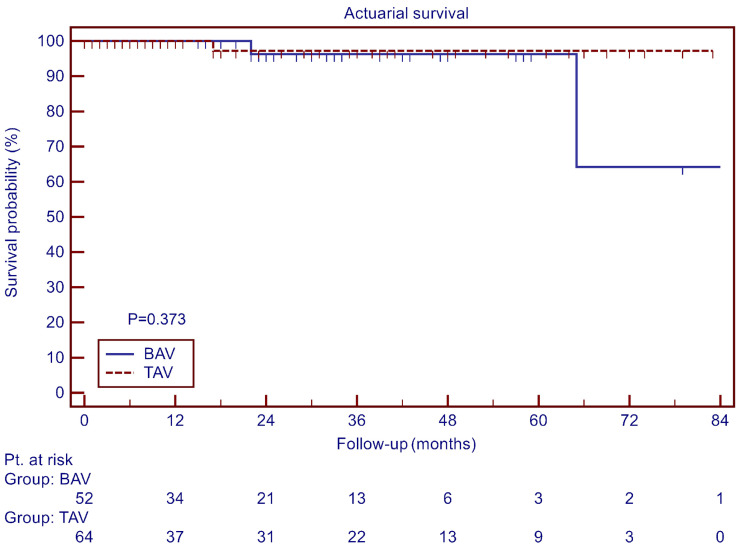
Overall survival.

Three patients died during follow-up, two of non-cardiac causes in the BAV group (3%) and one with sudden unexplained death in the TAV group (1%). Overall survival is depicted in Figure 1. The cumulative survival at 4 years was 96.3 ± 3.6% in the BAV group and 97.2 ± 2.7% in the TAV group (*p* = 0.373).

### 3.6. Aortic Valve Reinterventions and Aortic Regurgitation

**Figure 2:**
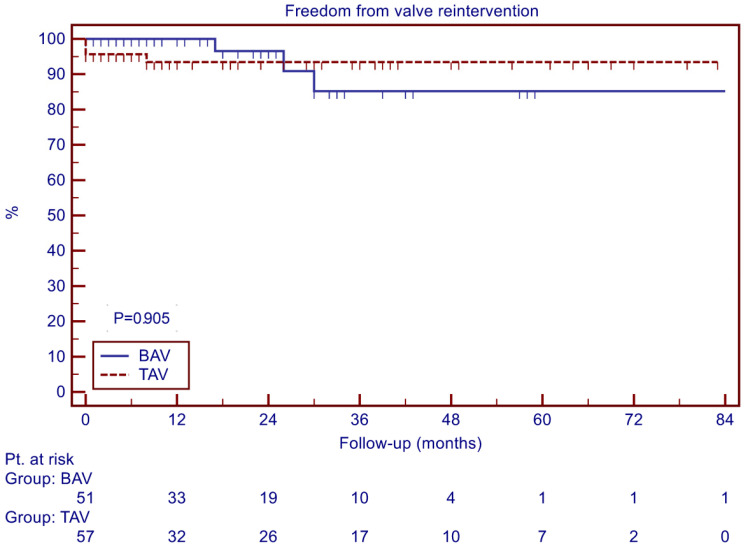
Freedom from AV reintervention.

There was a total of seven reinterventions on the aortic valve during follow-up and three additional cases of significant aortic regurgitation (AR > 2+). The freedom from AV reintervention at 4 years was 85.2 ± 8.1% for the BAV group and 93.4 ± 3.3% for the TAV group (*p* = 0.905, Figure 2). 

Four of the AV reinterventions were in the TAV group and three were in the BAV group. Most of the patients underwent VSRR + aortic valve repair with aortic annuloplasty, except in one case, in which tubular aorta replacement and aortic valve repair without aortic annuloplasty were performed. In two cases (both TAV group, Coroneo aortic annuloplasty performed), massive aortic regurgitation was diagnosed before discharge from the hospital. Intraoperatively, in the first case, NCC–RCC commissure rupture was found. In the second case, failure was caused by RCC leaflet damage due to contact with a subannular pledget, explained by excessive leaflet motion. The third patient (TAV group, Vacutek Gelweave aortic annuloplasty performed) presented 7 days after discharge from the hospital with pseudoaneurysm of the aortic root caused by laceration of the LVOT by the annuloplasty suture. Regarding a later onset of repair failure, four cases were detected. The first case (BAV group, Coroneo aortic annuloplasty performed) presented six months after the repair and suture line dehiscence between the LCC and RCC was observed. The second case required reoperation 2 years after the repair owing to native valve endocarditis (owing to aortic root rupture due to the aortic annuloplasty ring). Also, in a similar time period, in another patient (BAV group, Coroneo aortic annuloplasty performed), failure appeared due to a laceration of the aortic valve leaflet caused by the central placating suture of a fused cusp. Finally, the last case (TAV group, no aortic annuloplasty performed) presented with massive aortic regurgitation on a regular echocardiography examination 9 months after the primary operation and underwent a TAVI procedure soon after. Even though the valve could not be observed intraoperatively, failure was explained by postoperative dilatation of the aortic annulus as a consequence of lacking aortic annuloplasty. 

**Figure 3:**
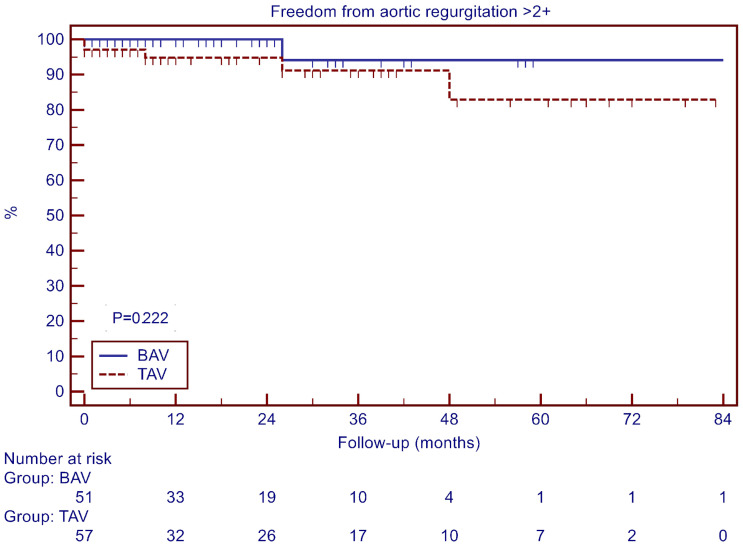
Freedom from aortic regurgitation > 2+.

The freedom from severe aortic regurgitation (AR > 2+) at 4 years was 94.1 ± 5.7% for the BAV group and 82.9 ± 8.9% for the TAV group (*p* = 0.222, Figure 3).

### 3.7. Echocardiography

Follow-up echocardiography showed a slightly higher mean AV gradient in the BAV group (10 ± 9 mmHg vs. 7 ± 6 mmHg, *p* = 0.009) as well as a lower coaptation height (9 ± 3 mm vs. 11 ± 5 mm, *p* = 0.024). There was no difference concerning left ventricular remodeling (expressed as delta LVEDD) between groups. There were no significant differences in other echocardiography data.

## 4. Discussion

Our study shows that surgical repair of BAV using root remodeling or isolated valve repair combined with calibrated extra-aortic annuloplasty can be performed with high reproducibility (shown as 100% success of initial operation) and low perioperative morbidity and mortality. The mid-term results at 4 years after repair expressed as freedom from aortic valve reintervention (85% for BAV, 93% for TAV group, *p* = 0.905) or freedom from severe aortic regurgitation (94% for BAV, 83% for TAV group, *p* = 0.222) show that BAV repair is at least comparable with the results of repair in the TAV group. The cumulative survival at 4 years was excellent in both groups (96% in BAV and 97% in the TAV group, *p* = 0.373). 

It seems that the slightly lower utilization of extra-aortic annuloplasty rings in the TAV group (89% vs. 100%) did not have a negative impact on the mid-term durability of repair. Although the mean postoperative gradient across the aortic valve is statistically higher in the BAV group (10 mmHg vs. 7 mmHg), it was not manifested in clinically important outcomes in the observed follow-up period.

To this day, there is scarce literature about the differences between BAV and TAV groups after valve repair and our results correspond to the results of several bigger studies with different surgical techniques applied. Aicher and colleagues showed excellent outcomes of root remodeling AV repair in a group of two hundred and forty-nine patients, with a freedom from severe AR at 5 years of 96% in the BAV group and 88% in the TAV group (*p* = 0.08), with only nine patients requiring reoperation (3.6%) [12]. Svensson et al. discovered in their cohort of 728 patients with BAV repair that at 5 years, 25% of patients had moderate (2+) and 24% severe AR (3+/4+), but the freedom from reoperation remained high (87%, 78%, and 64% at 5, 10, and 15 years, respectively) [13]. In the more recent report from the same group, the authors found that patients undergoing cusp repair combined with root procedures (mostly root reimplantation) were less likely to have a high postoperative AR grade and had a lower mean gradient and lower LV mass index over time compared with patients with isolated cusp repair without annular support [14]. The importance of annular stabilization was further shown by Lansac et al. in a group of 232 patients with valve repair with external aortic ring annuloplasty with a high freedom from severe postoperative AR at 7 years of 90.6% and no differences between BAV and TAV [15]. Ouzounian and colleagues showed superb results of valve-sparing root replacement using a reimplantation technique in a group of 333 patients at 5 years, with no valve reoperation in either BAV or TAV propensity-matched groups, but with a higher incidence of severe AR in the BAV group (3.9% vs. 1.2%) which was not statistically significant (*p* = 0.08) [16]. Gocol and colleagues showed considerably higher 5-year survival in the BAV than in the TAV group (97% vs. 80, *p* < 0.001) [17]. Rates of reintervention and AR recurrence were similar in both groups. Their spectrum of operation included both internal and external annuloplasty, as well as both root reimplantation and remodeling techniques when root replacement was needed. Survival differences were attributed to the younger age and less comorbidity in BAV patients.

However, there are some limitations to our study that must be considered. This research is a retrospective, observational analysis of single-center results prospectively collected through the AVIATOR database. The number of patients in both groups was limited and relatively small for accurate data analysis due to the vast heterogeneity of aortic valve pathology and multiple types of surgical procedures. This was somewhat mitigated by our adherence to standardized surgical techniques, measurement, and reasoning as previously described. Furthermore, the initial echocardiography examination and follow-ups were not consistently carried out by the same person, resulting in some measurements being incomplete or inconsistent. Moreover, this study’s limitations include a patient age range of 47–60 and a short follow-up period, limiting our insight into longer-term outcomes.

## 5. Conclusions

Based on our experience, we conclude that aortic valve repair is a safe and feasible strategy in patients with aortic regurgitation regardless of valve anatomy (BAV or TAV). Reproducible step-by-step procedure respecting individual anatomy and pathology, and especially the addition of calibrated external ring annuloplasty contributes to early and mid-term repair success. Long-term durability remains to be analyzed.

## Figures and Tables

**Table 1:** Preoperative patient characteristics.

Variables	Total, *n* = 132	BAV, *n* = 58	TAV, *n* = 74	*p* Value
Age (years)	54 ± 19	47 ± 18	60 ±14	<0.001
Gender, female	23 [17]	6 [10]	17 [23]	0.67
Height (cm)	178 ± 14	178 ± 14	178 ± 13	0.540
Weight (kg)	89 ± 22	90 ± 17	88 ± 11	0.936
BMI (kg/m^2^)	28.07 ± 6	27.77 ± 5.21	28.23 ± 6.34	0.473
NYHA				0.766
I	35 [27]	17 [29]	18 [24]	
II	82 [62]	35 [60]	47 [64]	
III	14 [11]	6 [10]	8 [11]	
IV	1 [1]	0	1 [1]	
Creatinine (mg/dL)	0.97 ± 0.26	0.99 ± 0.23	0.96 ± 0.28	0.681
EuroSCORE I (%)	5.80 ± 3.51	4.65 ± 2.11	7.20 ± 5.64	<0.001
EuroSCORE II (%)	2.20 ± 1.12	1.83 ± 0.85	2.33 ± 1.21	0.005
Arterial hypertension	102 [77]	38 [66]	64 [87]	0.006
Diabetes mellitus	3 [2.3]	1 [1.7]	2 [2.7]	1.000
Aortic dissection	5 [3.8]	2 [3.4]	3 [4.1]	1.000
Atrial fibrillation	10 [7.6]	3 [5.2]	7 [9.5]	0.512
COPD	2 [1.5]	0	2 [2.7]	0.504
Connective tissue disease				0.531
Yes	7 [5]	2 [3]	5 [7]	
Unknown	19 [14]	10 [17]	9 [12]	
Previous cardiac surgery	1 [1]	0	1 [1]	1.000
Recent MI	1 [1]	0	1 [1]	1.000
Aortic regurgitation			0.279	
0 (none or trivial)	3 [2]	1 [2]	2 [3]	
1 (mild)	8 [6]	6 [11]	2 [3]	
2 (mild to moderate)	23 [18]	7 [13]	16 [23]	
3 (moderate to severe)	51 [40]	22 [40]	29 [41]	
4 (severe)	41 [33]	19 [35]	22 [31]	
LVEF (%)	60 ± 9	60 ±13	60 ± 9	0.814
LVEDD (mm)	61 ± 12	61 ± 10	60 ± 12	0.605
LVESD (mm)	42 ± 11	42 ± 10	42 ± 12	0.947
Aortic annulus (mm)	28 ± 6	29 ± 5	28 ± 5	0.014
Sinuses diameter (mm)	44 ± 9	41 ± 6	46 ± 13	0.001
Sinotubular junction (mm)	40 ± 11	39 ± 10	42 ± 11	0.032
Ascending aorta (mm)	47 ± 15	45 ± 15	49 ± 18	0.059

Data expressed as *n* [%] or median ± IQR; MI—myocardial infarction; LVEF—left ventricular ejection fraction; LVEDD—left ventricular end-diastolic diameter; LVESD—left ventricular end-systolic diameter.

**Table 2:** Intraoperative patient data.

Variables	Total, *n* = 132	BAV, *n* = 58	TAV, *n* = 74	*p* Value
Type of repair				0.209
Isolated valve repair	20 [15]	13 [22]	7 [9]	
Partial root replacement (1–2 sinus) ± valve repair	4 [3]	2 [3]	2 [3]	
Tubular aorta replacement ± valve repair	22 [17]	8 [14]	14 [19]	
Valve-sparing root replacement ± valve repair	86 [65]	35 [60]	51 [69]	
Annuloplasty	124 [93.9]	58 [100]	66 [89]	0.009
External ring	121 [91.7]	58 [100]	63 [85]	0.009
Extra-aortic Coroneo Inc.	110 [91]	51 [88]	59 [94]	0.274
Dacron	11 [9]	7 [12]	4 [6]	
External ring size (mm)	27 ± 4	27 ± 3	27 ± 4	0.731
STJ ring	12 [9]	9 [15]	3 [4]	0.004
STJ ring size (mm)	29 ± 3	28 ± 3	29 ± 4	0.352
Cusp repair	115 [87]	56 [97]	59 [80]	0.003
Cabrol stitches	3 [2]	1 [2]	2 [3]	0.032
Graft size (mm)	28 ± 4	28 ± 2	28 ± 4	0.869
CPB (min)	138 ± 43	127 ± 40	145 ± 46	<0.001
CCT (min)	107 ± 26	104 ± 24	112 ± 32	0.004
Concomitant procedures	22 [17]	17 [23]	5 [9]	0.280
CABG	10 [8]	7 [9]	3 [5]	0.074
Aortic (hemi)arch replacement	11 [8]	9 [12]	2 [3]	0.082
Mitral valve procedure	4 [3]	1 [2]	3 [4]	0.089
Valve repair success	127 [96]	58 [100]	69 [93]	0.044

Data expressed as *n* [%] or median ± IQR; STJ—sinotubular junction; CPB—cardiopulmonary bypass; CCT—aortic cross-clamp time.

**Table 3:** Postoperative complications.

Variables	Total, *n* = 132	BAV, *n* = 58	TAV, *n* = 74	*p* Value
Reopening for bleeding	9 [7]	3 [5]	6 [8]	0.235
Permanent pacemaker	2 [1.52]	0 [0]	2 [3]	0.207
CVA (permanent)	1 [1]	0 [0]	1 [1]	0.183
CVA (transient)	1 [1]	0 [0]	1 [1]	0.183
Perioperative mortality	0 [0]	0 [0]	0 [0]	0.999

Data expressed as *n* [%]. CVA—cerebrovascular accident.

**Table 4:** Follow-up with echocardiographic data.

Variables	Total, *n* = 127	BAV, *n* = 58	TAV, *n* = 69	*p*-Value
Follow-up duration (d)	483 ± 946	479 ± 770	508 ± 946	0.527
Late mortality	3 [2]	2 [3]	1 [1]	0.435
AV reintervention	7 [6]	3 [5]	4 [6]	0.596
AV mean gradient (mmHg)	8 ± 7	10 ± 9	7 ± 6	0.009
Aortic annulus (mm)	26 ± 5	26 ± 5	26 ± 6	0.710
Sinuses diameter (mm)	32 ± 5	32 ± 5	32 ± 5	0.927
Sinotubular junction (mm)	29 ± 5	30 ± 4	29 ± 5	0.290
Ascending aorta (mm)	31 ± 5	32 ± 5	32 ± 5	0.148
LVEF (%)	60 ± 12	60 ± 10	56 ± 12	0.077
LVEDD (mm)	55 ± 9	54 ± 10	56 ± 9	0.169
LVESD (mm)	36 ± 9	36 ± 10	37 ± 9	0.217
Delta EF (%)	0 ± 11	0 ± 12	−2 ± 12	0.057
Delta LVEDD (mm)	−6 ± 7	−6 ± 7	−5 ± 7	0.587
Aortic regurgitation				0.003
0 (none or trivial)	72 [57]	43 [74]	29 [42]	
1 (mild)	33 [26]	12 [21]	21 [30]	
2 (mild to moderate)	19 [15]	3 [5]	16 [23]	
3 (moderate to severe)	2 [2]	0 [0]	2 [3]	
4 (severe)	1 [1]	0 [0]	1 [1]	
Aortic regurgitation > 2+	3 [2]	0 [0]	3 [4]	0.157
Coaptation height (mm)	10 ± 4	9 ± 3	11 ± 5	0.024
Effective height (mm)	13 ± 4	13 ± 3	14 ± 4	0.145

Data expressed as *n* [%] or median ± IQR. LVEF—left ventricular ejection fraction; LVEDD—left ventricular end-diastolic diameter; LVESD—left ventricular end-systolic diameter.

## Data Availability

Data are available on demand due to privacy protection.
